# Severe neonatal hyperparathyroidism: a case report

**DOI:** 10.1186/1687-9856-2015-S1-P88

**Published:** 2015-04-28

**Authors:** Pathikan Dissaneevate, Sakda Patarapinyokul, Voraluck Phatarakijnirund

**Affiliations:** 1 Department of paediatrics, Hat Yai Hospital, Hat Yai, Songkhla, Thailand; 2 Department of surgery, Hat Yai Hospital, Hat Yai, Songkhla, Thailand; 3 Department of paediatrics, Pramongkutklao Hospital, Bangkok, Thailand

## Aims

To report case presenting with neonatal severe hyperparathyroidism

Methods: A severe neonatal hyperparathyroidism was reviewed including demographic and clinical data.

## Results

A 6-day-old girl was referred from province hospital due to lethargy, poor feeding and irritability. On examination, there were no dysmorphic features and apart from mild tachypnea, systematic examinations were normal. Her chest X-ray was also normal. Calcium and parathyroid hormone (PTH) levels at presentation were 17.3 mg/dL and 1,776 pg/mL, respectively. Firstly, she was managed with cefotaxime for treatment of sepsis. She received intravenous hydration, diuresis, hydrocortisone but calcium and PTH levels were persistent high. Furthermore, pamidronate was introduced and calcium level was decreased to normal range. Unfortunately, 7-10 days after stop pamidronate, calcium and PTH levels were gradually increased. A sestamibi nuclear scan was performed and showed hyperfunction parathyroid nodule at upper portion of both thyroid lobes. At age of 60 days, this girl went to have operation with a total parathyroidectomy and autotransplantation, using one half of gland placed into the right groin. Serum calcium and PTH were return to normal after operation.

## Conclusions

Neonatal severe hyperparathyroidism is managed effectively with total parathyroidectomy and autotransplantation.

Written informed consent was obtained from the patient's parent or guardian for publication of this abstract and any accompanying images. A copy of the written consent is available for review by the Editor of this journal.

